# N-Linked Glycosylation in Chinese Hamster Ovary Cells Is Critical for Insulin-like Growth Factor 1 Signaling

**DOI:** 10.3390/ijms232314952

**Published:** 2022-11-29

**Authors:** Rupashree Salvi, Chandan Kumar, Krupanshi Brahmbhatt, Rambhadur Subedi, Susan Idicula-Thomas, Taruna Madan, Barnali Biswas

**Affiliations:** 1 Department of Innate Immunity, ICMR—National Institute for Research in Reproductive and Child Health, Mumbai 400012, India; 2 Biomedical Informatics Centre, ICMR—National Institute for Research in Reproductive and Child Health, Mumbai 400012, India

**Keywords:** N-glycosylation, Orbitrap Mass Spectrometry (MS), insulin-like growth factor 1 receptor, Ras GTPase activating Protein (IQGAP1), integrins, receptor tyrosine kinase, MAP kinases (MAPKs), protein phosphorylation

## Abstract

Cell surface proteins carrying N-glycans play important roles in inter- and intracellular processes including cell adhesion, development, and cellular recognition. Dysregulation of the glycosylation machinery has been implicated in various diseases, and investigation of global differential cell surface proteome effects due to the loss of N-glycosylation will provide comprehensive insights into their pathogenesis. Cell surface proteins isolated from Parent Pro^–5^ CHO cells (W5 cells), two CHO mutants with loss of N-glycosylation function derived from Pro^–5^ CHO (Lec1 and Lec4 cells), were subjected to proteome analysis via high-resolution LCMS. We identified 44 and 43 differentially expressed membrane proteins in Lec1 and Lec4 cells, respectively, as compared to W5 cells. The defective N-glycosylation mutants showed increased abundance of integrin subunits in Lec1 and Lec4 cells at the cell surface. We also found significantly reduced levels of IGF-1R (Insulin like growth factor-1 receptor); a receptor tyrosine kinase; and the GTPase activating protein IQGAP1 (IQ motif-containing GTPase activating protein), a highly conserved cytoplasmic scaffold protein) in Lec1 and Lec4 cells. In silico docking studies showed that the IQ domain of IQGAP1 interacts with the kinase domain of IGF-1R. The integrin signaling and insulin growth factor receptor signaling were also enriched according to GSEA analysis and pathway analysis of differentially expressed proteins. Significant reductions of phosphorylation of ERK1 and ERK2 in Lec1 and Lec4 cells were observed upon IGF-1R ligand (IGF-1 LR3) stimulation. IGF-1 LR3, known as Long arginine3-IGF-1, is a synthetic protein and lengthened analog of insulin-like growth factor 1. The work suggests a novel mechanism for the activation of IGF-1 dependent ERK signaling in CHO cells, wherein IQGAP1 plausibly functions as an IGF-1R-associated scaffold protein. Appropriate glycosylation by the enzymes MGAT1 and MGAT5 is thus essential for processing of cell surface receptor IGF-1R, a potential binding partner in IQGAP1 and ERK signaling, the integral components of the IGF pathway.

## 1. Introduction

Cell membrane proteins and receptors are heavily glycosylated, a process which plays key roles in many biological processes, such as embryonic development, tissue development, and homeostasis in multicellular organisms [1,2,3,4]. They receive signals from the surroundings which are transmitted to the intracellular compartments and finally translated into molecular and cellular processes required for cell survival [5,6]. N-glycosylation affects a variety of cell membrane proteins, including growth factor receptors [7]. Altered glycosylation of growth factor receptors is responsible for disturbed cellular signaling and aggressive phenotypes [8,9]. Mammalian cells with mutations in glycosylation enzymes affecting the glycosylation pathway are important to study as these mutations affect the structure, folding, transport, and function of the proteins [9,10,11,12]. Chinese hamster ovary (CHO) cells harboring a panel of glycosylation mutations have been identified using selection for resistance to the cytotoxicity of various plant lectins [13,14]. These CHO mutants have been previously characterized biochemically and genetically [14]. In this study, we used two CHO glycosylation mutant cells: Lec1 cells, which lack MGAT1 and thus cannot add a terminal *N*-acetylglucosamine residue to the lower-branch mannose of Man_5_GlcNAc_2_; and Lec4 cells, which lack MGAT5 and cannot add *N*-acetylglucosamine to the biantennary branch [15].

Dysregulation of N-glycosylation has been observed under pathological conditions such as inflammation and cancer progression [16,17,18]. Studies have demonstrated the direct impact of glycosyltransferases in the context of several diseases including metabolic diseases, congenital diseases, and cancer, and the potential for individualized approaches to diagnostics and treatment [19,20,21,22,23]. Much less is known about how defects in each N-linked glycosylation enzyme alter the maturation, stability, and function of glycoproteins and other cell surface proteins. Thus, understanding these effects will provide clues towards the multisystem pathogenesis of congenital disorders of glycosylation and more common diseases. Several studies have suggested that remodeling of the cell surface N-linked oligosaccharides plays an important role in cell adhesion to the extracellular matrix (ECM) in multiple cell lines [24,25,26]. Integrins are the major adhesion and signaling receptor proteins that play important roles in regulating the cytoskeleton and receiving signals from the ECM [27,28,29]. They can bypass the downstream cell adhesion and migratory pathways and modulate the cytoskeleton, thus regulating cell motility and migration [27,30]. Differential expression of integrins has been reported in different disease conditions [31,32]. Integrins are also one of the regulators of metastasis [33,34]. For instance, inducing the expression of the αVβ3 integrin subunit in cancer cell lines increases their metastatic potential [35].

The Insulin-like growth factor 1 (IGF-1) signaling pathway is important for growth, survival, and development [36]. The Insulin-like growth factor 1 receptor (IGF-1R) is a type I membrane protein found on plasma membranes that forms dimers upon ligand binding (IGF-1), thus triggering signaling cascades [37]. The extracellular domain (ectodomain) is heavily N-glycosylated and contains the ligand binding site [38,39]. The cytoplasmic domain contains the tyrosine kinase that is activated to self-phosphorylate specific Tyr residues and bind to intracellular proteins, resulting in activation of pathways of cell survival, growth, or apoptosis [40]. IGF-1R has been shown to be of critical importance for tumor development and tumor cell survival in various types of malignancies [41,42,43,44]. Studies have demonstrated that an adequate N-linked glycosylation of IGF-1R is required for its translocation to the cell surface in melanoma cells [41,45]. This raises the possibility of using glycosylation inhibitors as therapeutic agents against IGF-1R-dependent malignancies [41].

MGAT5 adds the GlcNAcβ-1-6 branch to complex N-glycans and is associated with cancers and tumor progression [46]. Previous studies have reported that overexpression of Mgat5 and N-glycosylation increases the growth factors (Epidermal growth factor, TGF, VEGF) by increasing the galectin-3 residency on the cell surface via binding to the polylactosamine sugars of receptors [47,48,49]. On the other hand, knockdown of MGAT5 showed no effect on Epidermal Growth Factor (EGF) binding to Epidermal Growth Factor Receptor (EGFR), but resulted in reduced EGF-promoted activation of focal adhesion kinase and attenuation of the invasive phenotype in breast carcinoma cells [39].

The Ras GTPase-activating-like protein IQGAP1 (IQ motif-containing GTPase activating protein) has been identified as a novel growth factor receptor interacting partner [50]. Several studies have previously suggested that IQGAP1 participates in signaling cascades that bind to and modulate the activity of receptors such as the estrogen receptor [50], platelet-derived growth factor receptor (PDGFR) [51], epidermal growth factor receptor (EGFR) [52], human epidermal growth factor receptor-2 (HER2) [53], and vascular endothelial growth factor receptor-2 (VEGFR2) [54]. Previous studies have also reported that IQGAP1 is a modulator for the MAPK signaling pathway [55]. To date, no evidence has been found as to whether IQGAP-1 can regulate IGF-1R signaling via MAPK. IQGAP1 is involved in cytoskeletal organization and signaling through regulation of a number of cellular functions including cell-to-cell adhesion, migration, and integration of complex signaling pathways within the cell [56,57]. IQGAP1 encodes a ras GAP-related protein containing the IQ motif [57,58,59]. Previous findings suggest that IQGAP1 silencing plays crucial roles in the apoptosis of HepG2 cells and lowers their proliferative and invasive capacities [60]. Studies have reported that IQGAP1 functions as a scaffold, assembling crucial components of both the PI3K–Akt and Ras–ERK pathways [61]. The Ras–ERK and PI3K–Akt pathways are key to the control of cell growth, survival, metabolism, and motility [62].

The aim of this study was to investigate the effects of the loss of Mgat1 (Lec1 cells) and Mgat5 (Lec4 cells) on cell surface proteins, including integrins and glycosylated proteins. We performed a high-resolution LCMS analysis of cell surface proteins isolated from W5 (control), and two CHO glycosylation mutants that were shown to lack N-linked carbohydrates, identified as Lec1 (Mgat1 null) and Lec4 (Mgat5 null) with Man_5_GlcNAc_2_Asn and GlcNAcbeta1,6 Man alpha l,6 branches respectively. We identified the as-yet unavailable differentially expressed cell surface proteins of CHO cells (W5, Lec1, Lec4). We hypothesize that inhibition of the N-linked glycosylation pathway in CHO cells due to the loss of Mgat1 and Mgat5 genes might result in the downregulation of glycoproteins, growth factor receptors, and their associated proteins. Previous studies have demonstrated that IQGAP1 binds to extracellular signal-regulated kinase (ERK) 2 and regulates growth-factor-stimulated ERK activity in breast cancer cells. No studies to date have been done to understand whether N-glycosylation determines IQGAP-1 levels and regulates ERK activity. In our study, using LCMS proteome analysis, we found less abundance of IGF-1R and IQGAP-1 at the cell surface in Lec1 and Lec4 cells. This initial finding led us to the question of whether IGF-1R regulates ERK signaling via its interaction with IQGAP-1 in W5 and Lec1/Lec4 cells. In this work, we stimulated the cells with a ligand of IGF-1R (IGF-1LR3) to enhance the expression of IGF-1R and check for ERK activity. We provide in silico evidence to suggest that IQ domain of IQGAP1 is a binding partner for the kinase domain of IGF-1R. In contrast, changes in surface expression of integrins in Lec1 and Lec4 cells as compared to W5 were also observed. Our data provide evidence that CHO cells with loss of initial branching (Lec1 cells) and latter branching (Lec4 cells) for N-glycans have altered integrin expression, reduced IGF-1R and IQGAP-1 cell surface expression, and reduced IGF-1-induced IGF-1R levels and ERK or tyrosine kinase activation.

## 2. Materials and Methods

### 2.1. Cell Lines and Cell Culture

The cell lines used were parent CHO (Pro^–5^) and glycosylation mutants derived from Pro^–5^ CHO cells [15,63,64]. The Pro^–5^ line lacking transcripts of Mgat1 was named Lec1, and that lacking Mgat5 was termed Lec4.

The cells were a kind gift from Dr. Pamela Stanley (Albert Einstein College of Medicine). CHO parent (W5), Lec1, and Lec4 cells were grown in monolayers in α-minimal essential medium containing nucleotides and ribonucleosides (Catalog no.11900-024, Gibco, Carlsbad, CA, USA) and containing 10% fetal bovine serum (Catalog no.10270-106, Gibco) at 37 °C in 5% CO_2_.

### 2.2. Preparation of Cell Surface Proteins Using N-Hydroxysulfosuccinimydyl-S,S-Biotin (NHS-SS-Biotin) Based Cell Surface Protein Biotinylation and Isolation Kit

The isolation of plasma membrane proteins was performed using the commercially available Pierce Cell Surface Protein isolation kit (Catalog no. 89881, Thermo Scientific). In brief, W5, Lec1, and Lec4 cells were seeded in T75 cm^2^ flasks and maintained for 16 h in complete medium until 90–95% confluence. Cells were washed once with ice-cold PBS, resuspended at a concentration of 10^7^ cells/mL in ice-cold 10 mL biotinylation mix (one 12 mg vial of NHS-SS-biotin dissolved in 48 mL ice-cold PBS, pH 8.0) immediately before addition to cells, and incubated for 30 min at 4 °C. The biotinylation reaction was quenched by the addition of 500 µL quenching solution. Labeled cells were scraped and the pellet was dispersed in 200 µL of lysis buffer and 0.1 mg/mL protease inhibitor (Catalog no. A32953, Thermo Scientific, Waltham, MA, USA). Sonication was performed on ice using medium power and the lysate was incubated for 30 min in ice followed by centrifugation for 2 min at 1000× *g*. Clarified supernatant was enriched using 250 μL Neutravidin agarose for 1 h at room temperature. Biotinylated proteins were eluted from beads by incubation for 1 h at room temperature in 1% (w/v) SDS, 50 mM DTT, 100 mM Tris-HCl. The final elute was collected by centrifugation at 1000× *g* for 2 min. Proteins were denatured by heating for 10 min at 95 °C, followed by mass spectrometry.

### 2.3. Processing of Plasma Membrane Proteins for Mass Spectrometry

Proteomic analysis was carried out using the label-free Orbitrap liquid chromatography high-resolution mass spectrometer at IIT Bombay, SAIF. Approximately 100 µg measures of proteins from W5, Lec1, and Lec4 cells were separated on 10% SDS-PAGE gel, trimmed, and sent for identification of proteins by MS.

### 2.4. Data Processing

The data analysis software used for identification of the membrane proteins was Thermo Proteome Discoverer 2.2. The data used were abundance-grouped values for individual proteins. Fold change was calculated by comparing abundance-grouped values of Lec1 and Lec4 cells with control W5 cells. Proteins which did not have abundance values in control (W5) data were not considered for analysis. Hierarchical clustering analysis was done using Hemi.exe1 to find groupings among the glycosylation-deficient cells and to assess the clustering between the proteins.

### 2.5. Bioinformatics Analyses

In silico analysis of the candidate membrane proteins involved in several biological pathways and processes was done using PANTHER, DAVID/GO classification, ingenuity pathway analysis (IPA), and gene set enrichment analysis (GSEA).

### 2.6. Western Blot Analysis

Western blotting was performed according to standard procedures. In brief, the proteins were extracted from individual cells in RIPA lysis buffer (Catalog no. 822721, MP Biomedicals) supplemented with protease inhibitor (Catalog no. A32953, Thermo Scientific). Protein concentration was determined using a BCA protein assay kit (Catalog no. A23225, Thermo Scientific), and whole lysates were denatured at 95 °C in 4X Sample buffer and separated on SDS–polyacrylamide gels. The separated proteins were then transferred to PVDF membrane and blocked in 10% BSA, followed by primary antibody incubation in 1% BSA overnight at 4 °C and secondary antibody incubation in 1% BSA, and then imaging of the membranes was performed. Primary antibodies were IGF-1R (Catalog no. SAB4300359, Sigma-Aldrich, St. Louis, MO, USA), ERK1/2 (Catalog no. 4696S, Cell Signaling Technology, Danvers, MA, USA), Phosphorylated ERK1/2 (pERK1/2) (Catalog no. 4370S, Cell Signaling Technology), IQGAP1 (Catalog no. 20648, Cell Signaling Technology), and GAPDH (Catalog no.2118S, Cell Signaling Technology). Secondary antibodies used were HRP-conjugated anti-rabbit (Catalog no. Ab205718, Abcam, Cambridge, UK) and anti-mouse (Catalog no. 31430, Invitrogen, Waltham, MA, USA). Densitometry analyses of the bands were performed with ImageJ software (Version 1.53k).

### 2.7. In Silico Interaction Analysis of IGF-1R and IQGAP1

Blind docking was performed to assess the interaction between the IQ domain of IQGAP1 and kinase domain of IGF-1R. The structural coordinates of the IQ domain of IQGAP1 were modeled using the SWISS-MODEL server [65], and the kinase domain was retrieved from PDB (PDB ID: 1JQH). The two structures were docked using (a) the ZDOCK module of Discovery Studio 2021, (b) ClusPro 2.0 [66], and (c) the PatchDock server [67]. The poses obtained from ZDOCK and PatchDock were further refined using RDOCK and Fire Dock, respectively. The top ranked docked complex was retrieved from each docking algorithm, and visualized for binding sites and interacting residues using DS Visualizer.

### 2.8. ELISA Analysis

We used a colorimetric integrin-mediated cell adhesion array kit (ECM530, Merck) to perform the ELISA assay. Approximately 2 × 10^6^ cells/mL of each cell type (W5, Lec1, and Lec4 cells) were scraped with PBS containing 2.5 mM EDTA. The cells were washed three times with PBS to remove dead cells. Cells were then resuspended in the assay buffer provided. Next, 100 µL of this suspension was added to each coated well of the kit and incubated for 2 h at 37 °C in a 5% CO_2_ humidifier chamber. Unbound cells were washed with assay buffer. Next, 100 µL of cell stain solution was added to each well, incubated for 5 min, and removed, and cells were gently washed with distilled water. Finally, 100 µL of extraction buffer was added and incubated for 10 min on a shaker, and absorbance was determined at 540 nm.

### 2.9. Stimulation with IGF-1R Ligand

Control (W5) and glycosylation-deficient cells (Lec1 and Lec4) were plated at a density of 0.5 × 10^5^ cells/mL in MEM medium for 24 h in six-well plates. The cells were stimulated with and without IGF-1 LR3 (10 µg/mL) in MEM medium without serum for 10 min. The cells were washed with PBS and trypsinized and centrifuged at 10,000 rpm for 10 min.

### 2.10. Quantitative Real-Time PCR (qRT-PCR)

Total RNA was extracted using Trizol reagent (Catalog no 15596026, Invitrogen). The cDNA was generated using a first-strand cDNA synthesis kit (Catalog no K1632, Thermo Scientific), using 2 µg RNA and oligo(dT) primers. The qRT-PCR was performed using Power SYBR Green Supermix (Catalog no A25742, Thermo Scientific) on a CFX96 Touch Real-Time PCR Detection System (Bio-Rad, Hercules, CA, USA). All analyses were performed in duplicate on two independent RNA preparations and values were normalized to *Beta-Actin* and *Gapdh* using the 2^−ΔΔC^_T_ method. Gene-specific primers were designed using NCBI Primer-BLAST to amplify a region of 150–200 base pairs.

### 2.11. Statistical Analysis

GraphPad Prism 7.0 (GraphPad Software, Inc., La Jolla, CA, USA) was used for statistical analyses. All data were statistically analyzed using one-way ANOVA with a Bonferroni correction, followed by Fisher’s exact test for comparison of two groups. All values are depicted as mean ± standard deviation and are considered significant if *p*  <  0.05.

## 3. Results

### 3.1. Identification of Differential Protein Abundance in W5, Lec1, and Lec4 cells

**Figure 1:**
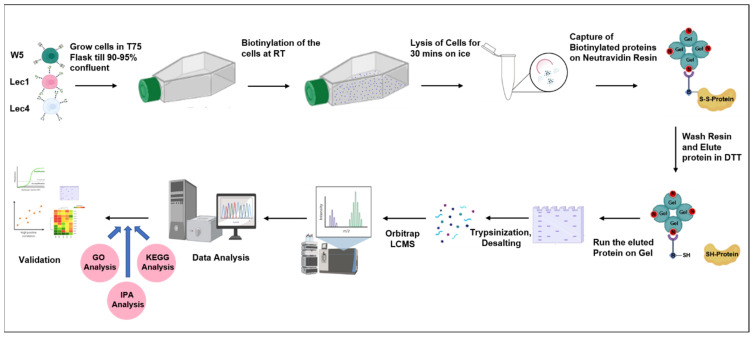
Workflow representation of the analysis of W5, Lec1, and Lec4 cells to identify cell surface proteins, run on HR–LCMS.

To identify cell surface proteins which are differentially regulated due to mutation in the key N-glycosylation enzymes (Mgat1 and Mgat5), label-free mass-spectrometry-based proteomics was performed to quantify altered amounts of protein in W5, Lec1, and Lec4 cells. The experimental strategy is depicted in Figure 1. Trypsin digestion was employed for sample preparation in three biological replicates and samples were subjected to a high-resolution liquid chromatograph mass spectrometer (HR-LCMS Orbitrap) for identification of proteins. The resulting data were validated against a *Mus musculus* proteome database for peptide/protein identification. The total ion chromatogram of the individual cells (W5, Lec1, and Lec4) on HR-LCMS, where total LC gradient time was 60 min) is shown in Supplementary Figure S1. In this study, a comprehensive analysis was performed using the HR- LCMS Orbitrap and Thermo Scientific Proteome Discoverer version 2.2. with the Uniprot (*Mus musculus*) database.

**Figure 2:**
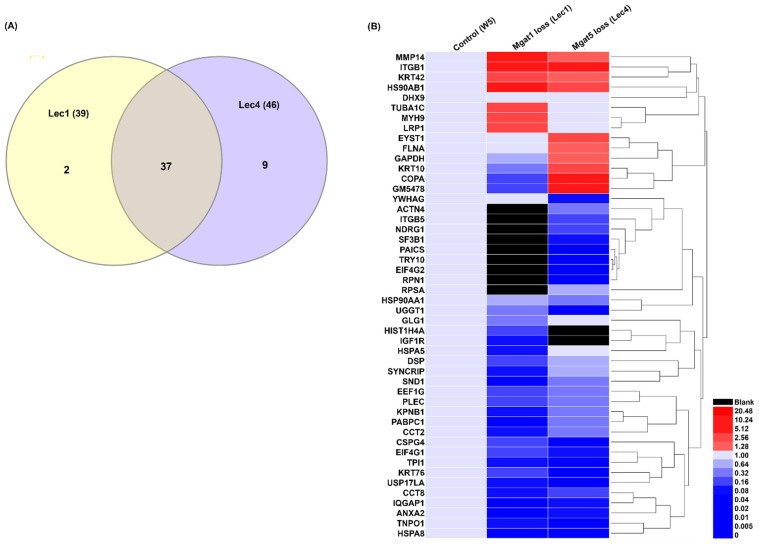
Identification of differentially expressed proteins from control Chinese hamster ovary (CHO) designated as W5, Lec1, and Lec4 cells using high-resolution liquid chromatograph mass spectrometry. (**A**) Venn diagram showing the overlapped proteins, indicating 37 common proteins between Lec1 and Lec4 cells; 9 proteins were present in Lec4 cells and 2 proteins were present in Lec1 cells. Venn diagram drawn using Bioinformatics and Evolutionary Genomics web tool (https://bioinformatics.psb.ugent.be/webtools/Venn/) (accessed on 23 December 2021) (**B**) Heat map comparative proteomic profile among control Chinese Hamster Ovary (CHO) W5, Lec1, and Lec4 cells, generated using Hemi.exe version 1.0 software. Color bar showing ranges of blue and red color indicates downregulation to upregulation of abundance values for each protein (scale is 0.00 up to 20.00). Black bar indicates no intensity abundance values for protein in cells.

To minimize the risk of false positive results, only proteins with a false discovery rate (FDR) or experimental q value less than 0.1% and with at least two unique peptides per protein were selected. To make the study more stringent, the proteins which did not have intensity values for control cells (W5) were not considered. A total of 48, 39, and 46 proteins were identified in W5, Lec1, and Lec4 cells, respectively. This is illustrated in a Venn diagram (Figure 2A). The Venn diagram was drawn using the Bioinformatics and Evolutionary Genomics web tool (https://bioinformatics.psb.ugent.be/webtools/Venn/) (accessed on 23 December 2021). Yellow color represents Lec1 cells and blue color represents Lec4 cells in comparison to W5 cells. We found 37 common proteins between Lec1 and Lec4 cells. We also found 2 proteins which were exclusive for Lec1 cells and 9 proteins which were exclusive for Lec4 cells. Differentially expressed proteins for W5, Lec1, and Lec4 cells, with a cut off of 1.5-fold increase and 0.77-fold decrease, are illustrated in a heat map (Figure 2B). The numbers of differentially expressed proteins that were detected in Lec1 and Lec4 cells were 44 and 43, respectively. The list of identified proteins, including the relative number of peptides of each protein and the abundance value, is given in Table 1. Heat maps were generated to show the hierarchical clustering of proteins displayed, confirming three distinct groups of control, Lec1, and Lec4. The up- and downregulated proteins in each group are shown in the heat map (Figure 2B). Overall, these proteins were involved in cytoskeleton remodeling, cell adhesion, growth factor signaling, and cell migration. The proteomic results indicated differential abundance of proteins involved in chaperone-related activity (HSP90AB1, HSP90AA1, CCT2, CCT8); alteration in cytoskeleton proteins (DSP, PLEC, TUBA1C); transport factors such as importin, exportin, and transportin (KPNB1, COPA, TNPO1); microtubule-associated proteins (KRT42, TUBA1C, KRT10, KRT76); integrins (ITGB1, ITGB5); GTPase activating protein (IQGAP1); helicase (DHX9); nuclease (SND1), glucosyltransferase (UGGT1); and Golgi glycoprotein (GLG1) (Table 2). Reduction of MGAT-1 and MGAT-5 in Lec1 and Lec4 cells, respectively, had an effect on the cell surface expression of integrin subunits as determined via LCMS analysis. Proteins enriched on the cell surface of Lec1 and Lec4 cells revealed altered integrin expression patterns, particularly for integrin β_1_ and β_5_, as compared to control cells (W5 cells) (Table 2). This might suggest that with loss of glycosylation, integrins were able to adhere to the extracellular matrix proteins. Previous studies indicate that N-glycan alterations observed on integrin subunits can influence integrin affinity for ligands and therefore regulate cell function towards a malignant phenotype.

### 3.2. Validation of the Proteomics Result

To confirm the HR-LCMS data for upregulated proteins (ITGB1, MMP14) and downregulated proteins (HSPA8, HSPA5, UGGT1, IQGAP1) in Lec1 and Lec4 cells as compared to control, q-PCR analyses were performed to determine the transcript levels for the genes. Real-time PCR analysis results for the upregulated and downregulated genes are shown in Supplementary Figure S2. The primers used for amplification of each gene of interest are shown in Table 3. The Ct (cycle threshold) values obtained for each genes were normalized to housekeeping genes (*Gapdh* and *Beta-Actin*) and the fold change for each gene was calculated as 2^ddCT^ = control gene-target gene. The fold change for upregulated and downregulated genes in Lec1 and Lec4 cells as compared to W5 or control (fold change = 1) were consistent with the proteomics data for the genes analyzed, and so the reliability of the sequencing is indicated (Supplementary Figure S2).

### 3.3. Functional Annotation and Enrichment of Differentially Expressed Proteins

**Figure 3:**
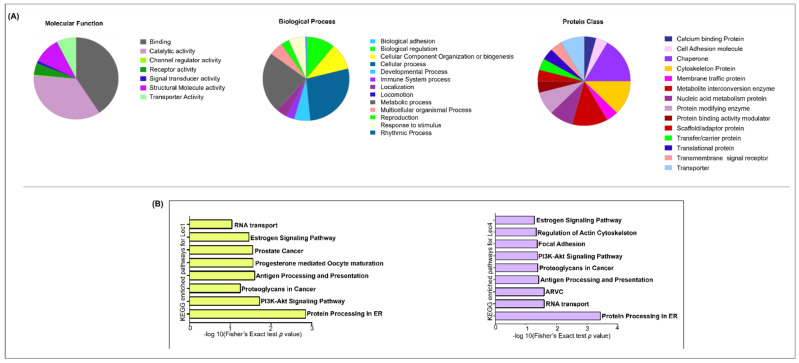
Functional annotation of differentially expressed proteins was analyzed via Protein Analysis through Evolutionary Relationships (PANTHER) and Database for Annotation, Visualization and Integrated Discovery (DAVID). (**A**) Molecular function, biological process, and protein class results of 37 dysregulated proteins in W5, Lec1 and Lec4 cells summarized in a pie chart using PANTHER; (**B**) biological processes in Lec1 and Lec4 based on the 44 and 43 differentially expressed proteins, depicted in a bar graph according to DAVID.

Gene Ontology analysis was performed to determine the potential biological relevance of the differentially expressed proteins. PANTHER analysis was performed according to ‘molecular functions’, ‘biological processes’, and ‘cellular components’. The most prominent GO terms involved in molecular function from Lec1 cells showed an enrichment in protein binding activity (51%), catalytic activity (25%), structural activity (5.12%), transporter activity (5.12%), molecular regulatory and transducer activity (2.56%), and translational activity (2.56%). In the biological process category, the most prominent processes included cellular response (58.97%), metabolic process (25.64%), response to stimulus (23.07%), biological regulation and localization (20.51%), developmental process (7.69%), signaling (7.69%), multicellular organismal process (7.69%), biological adhesion (5.12%), and locomotion (3.56%). In the ‘protein class’ classification, most cell surface proteins were related to chaperone activity (10.2%), cytoskeleton proteins (7.6%), metabolite interconversion (7.69%), transporter (5.12%), and protein-modifying enzymes (5.12%) (Figure 3A, Supplementary Table S1).

KEGG pathways associated with the identified surface proteins were analyzed using the DAVID classification pathway. In the KEGG enrichment results with a significance less than 0.2, eight pathways were detected to be significantly enriched in Lec1 cells. The key pathways in Lec1 cells were Protein processing in ER (*p*-value = 1.4 × 10^−2^), PI3K–AKT pathway (*p*-value = 1.9 × 10^−2^), and Signaling of proteoglycans in cancer (*p*-value = 2.2 × 10^−2^). In Lec4 cells, a total of nine key pathways were found through KEGG analysis. The top pathways observed were Protein processing in ER (*p*-value = 3.6 × 10^−4^), RNA transport (*p*-value = 2.5 × 10^−2^), and Arrhythmogenic right ventricular cardiomyopathy (ARVC) (*p*-value = 2.6 × 10^−2^) (Figure 3B, Supplementary Table S2). We speculate that glycosylation of the membrane proteins probably regulates protein processing in the ER in order to adapt to the changes in glycan conformation of the required proteins.

### 3.4. Interaction Networks of Surface Proteins Associated with Lec1/Lec4 Cells

**Figure 4:**
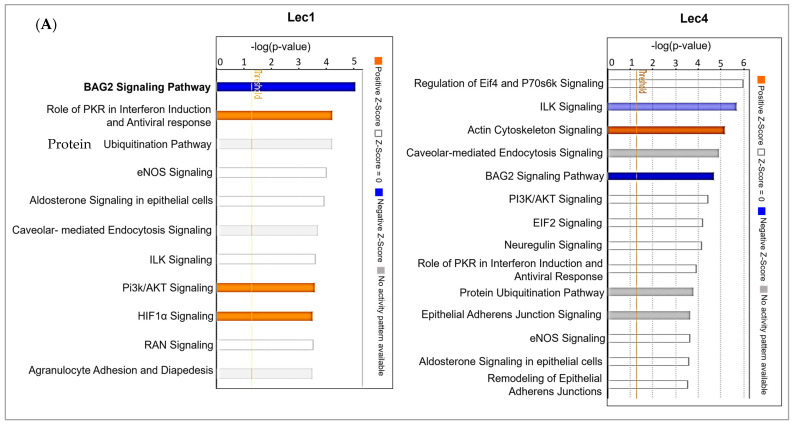

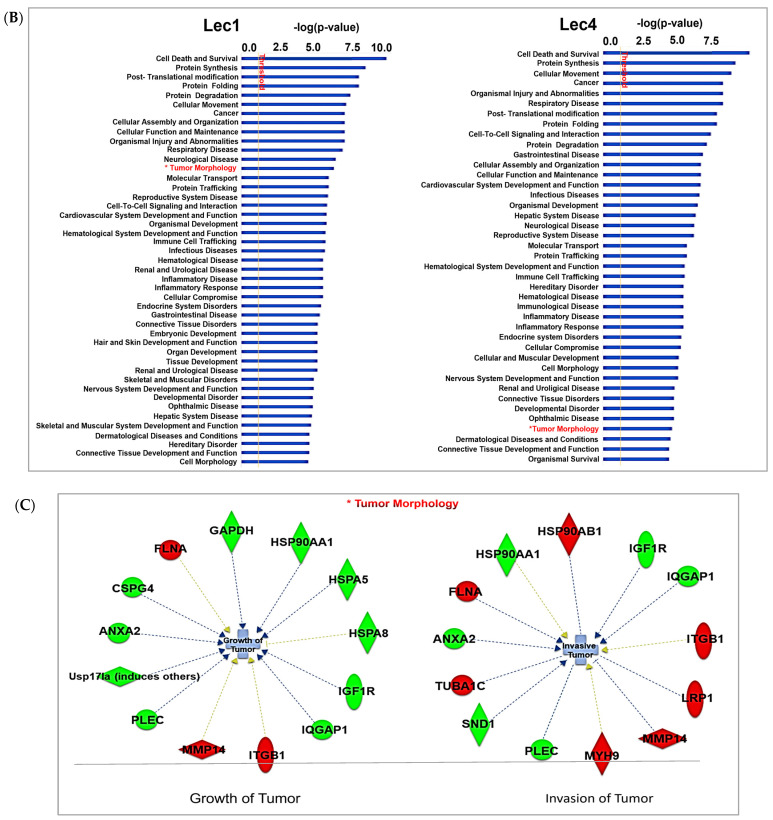
Ingenuity Pathway Analysis (IPA, https://www.qiagenbio-informatics.com/products/ingenuity-pathway-analysis/ (accessed on 30 April 2021)) of the differentially expressed proteins revealed top canonical pathways and downstream disease and biological pathways between control CHO and Lec1/Lec4 cells. (**A**) Top 11 enriched canonical pathways according to Ingenuity Pathway Analysis core analysis are shown here. The -log (*p*-value), z-score, and ratio of the top 11 significantly activated canonical signaling pathways are listed. A scale from light blue to dark blue indicates the level of activation of the canonical signaling pathways. The straight orange vertical line running through the bars is the *p*-value threshold for a particular pathway’s enrichment. The horizontal axis is the –log (*p* value) and the vertical axis represents the given pathways. (**B**) Bar graphs illustrate the disease and disorder enrichment in Lec1 and Lec4 based on the “*p*-value of overlap” of the proteins in our dataset relative to the IPA’s predefined categories. The -log values (*p*-values) of the top 45 significantly involved diseases and functions are listed for Lec1 and Lec4 cells. (**C**) Significant upregulated and downregulated proteins which influence tumor growth and invasion from the list of pathways involved in diseases and disorders in Lec1 cells are illustrated.

The differentially expressed proteins in Lec1 and Lec4 cells were uploaded to the Ingenuity Pathway Analysis (IPA) database in order to analyze biological events and generate possible regulatory networks. Using IPA, canonical pathways were determined and relationships between the most significant differential expressed proteins in Lec1 and Lec4 cells were examined. A total of four statistically significant pathways were obtained based on the z-scores. The top canonical pathway in Lec1 cells was the BAG2 signaling pathway (Figure 4A). A comparison of canonical pathways between Lec1 and Lec4 cells showed them to be distinct. The significance value is a measure to understand the likelihood of genes required for a particular pathway.

The BAG2 signaling pathway was also among the top five canonical pathways in Lec4 cells. The other top signaling pathways in Lec4 cells were the EIF4 and p7056k pathways (Figure 4A). The role of loss of N-glycosylation in diseases and cellular functions was then determined by applying the −log (*p* value) > 4 threshold in the IPA system.

The top categories of diseases and functions associated with dysregulated proteins in Lec1 and Lec4 cells are depicted in Figure 4B. A histogram containing a representative classification of diseases and functions is shown. Mgat1 and Mgat5 were found to serve important roles in a number of cellular functions, including cell death and survival, protein synthesis, cellular movement, post-translational modification, protein degradation, and cancer. One of the top hits in diseases and disorders was associated with tumor morphology (Figure 4B, marked in red). The top networks of interest related to the development of tumor cells were tumor growth and invasion, as shown in Figure 4C. The proteins are shown as either red or green, representing higher or lower abundance in Lec1 cells, respectively. The dashed lines represent indirect relationships and blue lines signify inhibition of tumor growth and invasiveness in Lec1 cells (Figure 4C).

### 3.5. Pathway Analysis

Network analysis was used to show the interactions of the molecules included in our dataset as well as molecules involved in the associated pathways using web-based knowledge. This study identified four statistically significant molecular networks from Lec1 and Lec4 cells (Supplementary Table S3). Each network corresponded to a significant biological function.

In Lec1 cells, the highest ranked network (network 1) had a score of 28; it contained 35 nodes and was involved in post-translational modification, protein degradation, and protein synthesis. This network (network 1) included 17 molecules (CCT2, EIF4G1, HSP90AA1, TNPQ1, CCT8, HSP90AB1, TUBA1C, EEF1G, HSPA8, PABPC1, YWHAG, and GAPDH) from the Lec1 dataset (Table 2).

**Figure 5:**
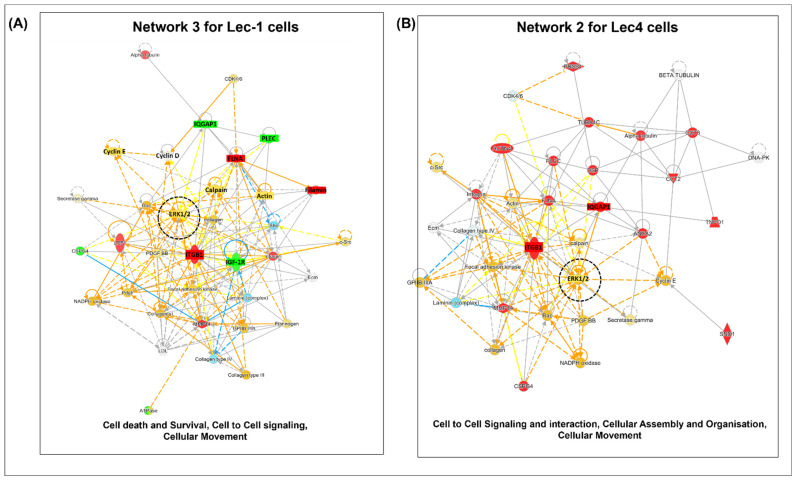
Top networks in Lec1 and Lec4 cells derived from Ingenuity Pathway Analysis. Upregulated genes are labeled in red, while downregulated genes are labeled in green. Ellipses represent transcription regulators, rhombuses represent enzymes, trapezoids represent transporters, double circles represent a complex/group, and circles represent others. (**A**) In Lec1 cells, network 3 was associated with ERK1/2. Focal adhesion kinase, Ecm, fibrinogen, collagen, actin, filamin, and Plec integrins regulate ERK molecules. (**B**) In Lec4 cells, network 2 was associated with cell-to-cell signaling and interaction, cellular assembly and organization, and cellular movement. The central molecules were ERK and integrin, ITGB1, ERK, tubulin, IQGAP1, laminin, collagen, FLNA, and Plec.

One of the interesting network maps, ranked at number 3 with a score of 17 and involving eight molecules (IGF-1R, FLNA, CSPG4, IQGAP1, LRP1, ITGB1, MMP14, and PLEC), affects cell death and survival, cell to cell signaling, and cellular movement (Supplementary Table S3). The associated interaction network map involving these molecules is shown in Figure 5A. The proteins marked in red or green were upregulated or downregulated, respectively, influencing the pathway. The blue and orange dotted lines indicate predicted inhibition or activation of the pathway. The majority of the molecules in network 3 were networked to the extracellular signal-regulated kinase/mitogen-activated protein kinase (ERK/MAPK) pathway. For cellular movement, the hub molecules from our dataset included FLNA, integrin (ITGB1), CSPG4, LRP1, PLEC, and MMP14; for cell-to-cell signaling and interaction, IGF-1R and IQGAP1 were involved (Supplementary Table S3, Figure S5A). Network 2, which had a score of 25 and involved 11 molecules, had an impact on cellular function and maintenance, cell death, and survival. ERK1/2 was found to be very strongly interconnected across all the nodes in this network.

For Lec4 cells, as shown in Figure 5B, the highest ranked network (score 38) was found to mainly affect post-translational modification, protein folding, and protein synthesis, and involved 16 molecules (EEF1G, EIF4G2, HSPA90AB1, LRP1, SF3B1, HSPA5, NDRG1, UGGT1, HSPA8, YWHAG, DHX9, EIF-4G1, GAPDH, HSP90AA1, PABPC1, and RPSA). Network 2, with a score of 35, affected cell-to-cell signaling and interaction, cellular assembly and organization, and cellular movement, and involved 15 molecules (CCT8, CSPG4, PLEC, SND1, ACTN4, MMP14, PRSS1, TNPO1, FLNA, IQGAP1, TUBA1C, ANXA2, CCT2, DSP, and ITGB1). ERK1/2, the central molecule in the network, showed the highest number of edges in network 2. This higher-order analysis from network 2 illustrates that integrin signaling, Rac, and IQGAP1-dependent (IQGAP1 is the GTP binding protein in oncogenesis) activation of MAPK/ERK is a critical event in the loss of N-glycosylation in Lec4 cells (Figure 5B).

We used gene set enrichment analysis (GSEA), a pathway enrichment method that evaluates proteomics data at the level of gene sets. GSEA analysis also revealed that the dysregulated proteins in Lec1 and Lec4 cells were associated with 15 and 12 pathways, respectively, with normal enrichment scores of either −1 or +1. The pathways were the insulin IGF pathway–protein kinase B signaling cascade, glycolysis, cytoskeleton regulation by Rho GTPase, MAP kinase cascade, and the integrin signaling pathway (Supplementary Table S4).

### 3.6. Integrin Regulation

**Figure 6:**
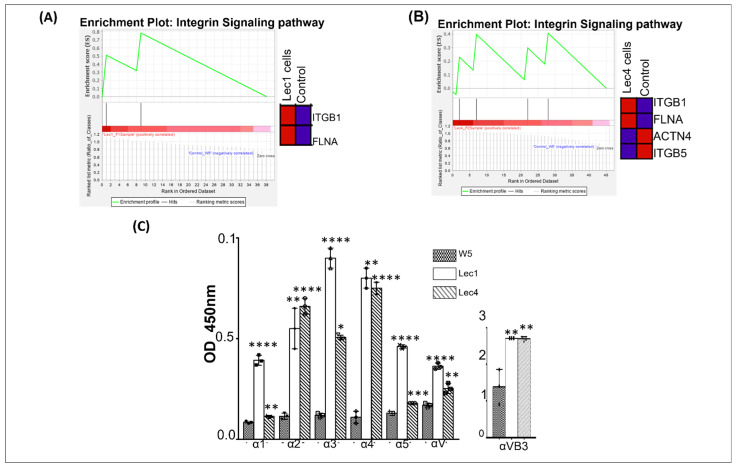
Analysis of cell surface integrins for differentially expressed proteins in Lec1 and Lec4 cells via GSEA analysis and ELISA assay. GSEA enrichment plots of gene clusters that were enriched in (**A**) Lec1 and (**B**) Lec4 cells in the integrin signaling pathway. Heat map shows the genes that regulate this pathway; red color indicates upregulation and blue color indicates downregulation. (**C**) Binding assay for integrins (α1, α2, α3, α4, α5, αvβ3) in control (W5), Lec1, and Lec4 cells detected by ELISA assay. The cells were co-incubated with the corresponding integrins in the ELISA-coated microwell plates and integrin binding to the cell surface was detected. Cell attachment was analyzed by obtaining optical density at 450nm. Negative control was done without coated antibody to calculate the amount of integrins expressed in each cell type (W5, Lec1, and Lec4). Data are shown as means S.D. of triplicate experiments. * *p* value < 0.05, ** *p* value < 0.01, *** *p* value < 0.001 and **** *p* value < 0.0001.

Gene set enrichment analysis (GSEA) showed upregulation of the integrin signaling pathway, which includes integrin beta 1 (ITGB1) and filamin A (FLNA) proteins in Lec1 cells and integrin beta 1 and 5 (ITGB1 and ITGB5), actin 4 (ACTN4), and filamin A (FLNA) in Lec4 cells (Figure 6A,B). Heat mapping showed proteins involved in the pathway. The normalized enrichment score was 1 and the FDR q value was 1, which was nonsignificant (Figure 6A).

To evaluate the loss of β1,2-GlcNAc branched N-glycans/GLNacTI (Lec1) or β1,6-GlcNAc branched N-glycans/GlcNAcTV (Lec4) on integrin expression, we evaluated alpha integrins using ELISA (Figure 6C). We quantified the optical density ratio of integrins (α1, α2, α3, α4, α5, αV, αVβ3) in the cell lysates from control, Lec1, and Lec4 cells at 450nm. The profile for surface integrins in Lec1 and Lec4 cells increased significantly compared to that of control cells (W5). We found that the average increase for alpha integrins (α1, α2, α3, α4, α5, αV) in Lec1 cells was 0.59, and in Lec4 cells was 0.36. Moreover, one of the most interesting subunits, αVβ3, increased 1.31-fold in Lec1 and 1.30-fold in Lec4 cells as compared to W5 cells (Figure 6C). These results indicate that the removal of β1,2-GlcNAc branched or β1,6-GlcNAc branched branched N-glycans might have an effect on integrin expression and regulation.

### 3.7. Insulin Growth Factor I Receptor Expression

**Figure 7:**
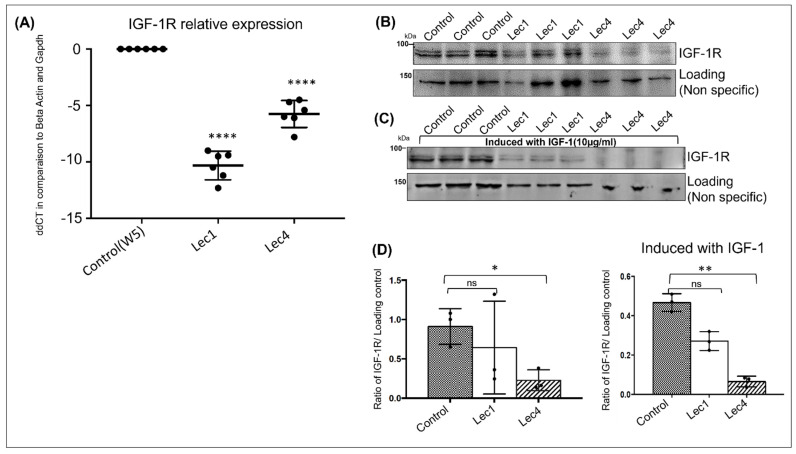
Insulin-like growth factor-1 receptor (IGF-1R) transcript and protein expression levels in W5, Lec1, and Lec4 cells. Cells were incubated in serum-free media for 12 h and were unstimulated or stimulated with IGF-1 ligand for 10 min. (**A**) qRTPCR analysis of total IGF−1R transcript levels in W5, Lec1, and Lec4 cells compared to housekeeping genes *beta actin* and *Gapdh*, estimated by ddCT. **** *p* value < 0.0001. (**B**) Cells were incubated in serum-free media for 12 h; protein lysates were analyzed for IGF-1R using Western blot and a nonspecific band was used as loading control. (**C**) Cells were stimulated with IGF−1R ligand (IGF−1LR3) for 10 min. Protein lysates were analyzed by WB for IGF-1R and a nonspecific band was used as loading control. (**D**) Signals were quantified by densitometry, normalized to the nonspecific band, and expressed as ratio of IGF1-R to nonspecific for control (W5), Lec1, and Lec4 cells. Data correspond to mean ± SEM from three independent experiments. * *p* value = 0.05, ** *p* value < 0.01, and ns = nonsignificant.

Proteomic analysis by LCMS revealed reduced insulin growth factor I receptor (IGF-1R) protein levels in Lec1 cells (0.85) and undetectable levels in Lec4 cells as compared to W5 cells (Table 1). This reduction at the cell surface could have arisen due to decreased expression of the receptors, impaired folding or transport due to loss of N-glycans, or other indirect effects on enzymes or proteins that process their proprotein forms to mature receptor subunits. We also detected the transcript levels of IGF-1R in control (W5), Lec1, and Lec4 cells using qRT-PCR. The mRNA levels for Lec1 and Lec4 cells, as shown by ddCT (delta-delta Ct method), were reduced 11-fold and 6-fold, respectively, compared to control (Figure 7A).

Western blot analysis was also performed to understand the protein levels of IGF-1R in W5, Lec1, and Lec4 cells (Figure 7B). We observed a reduction in the levels of IGF-1R (molecular weight 95 kDa) in Lec1 cells and a significant reduction in Lec4 cells as compared to controls (Figure 7D, left panel). Taken together, we conclude that there was a marked decrease of IGF-1R transcript and protein levels in Lec1 and Lec4 cells.

To further understand the regulation of the IGF-1 signaling pathway by IGF-1R with changes in N-glycosylation, we stimulated the cells with a previously standardized dose of IGF-1R ligand (IGF-1LR3, 10 μg/mL for 30 min). In the control (W5), IGF-1R receptor was present in its matured form at 95kDa and, after induction, the amount of IGF-1R decreased further in Lec1 and Lec4 cells (Figure 7C,D, right panel). Loss of N-glycans on the IGF-1R receptor significantly reduced the mature form in Lec4 cells without stimulation (*p* < 0.05) and with induction (*p* < 0.01). We also observed a reduction in Lec1 cells, although the change was nonsignificant compared to control cells (Figure 7D).

### 3.8. Effect of Altered IGF1-R on Its Downstream Molecules/Pathways

**Figure 8:**
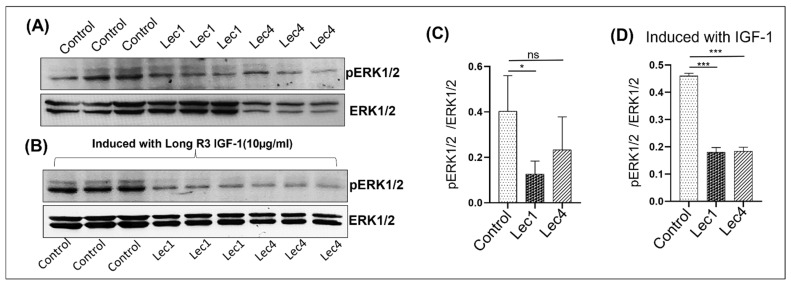
IGF-1 induced IGF-1R-mediated signaling activation. (**A**) Cells were incubated in serum-free medium for 12 h. Protein lysates were analyzed by WB for phosphorylated ERK 1/2(pERK1/2) and ERK1/2 as a loading control. (**B**) Cells were then treated with IGF-1 ligand (10 ug/mL) for 30 min. Protein lysates were analyzed by WB for phosphorylated ERK 1/2(pERK1/2) and ERK1/2 as a loading control. (**C**) Signals were quantified by densitometry, normalized to ERK1/2, and expressed as the ratio of (pERK1/2)/(ERK1/2) in W5, Lec1, and Lec4 cells without stimulation with ligands. (**D**) Signals were quantified by densitometry, normalized to ERK1/2, and expressed as the ratio of (pERK1/2)/(ERK1/2) in W5, Lec1 and Lec4 cells with stimulation with ligands. Data correspond to the mean ±SEM. * *p* value = 0.05, *** *p* value < 0.001 and ns = nonsignificant.

To further understand the regulation of the IGF-1 signaling pathway and the downstream signaling events that occur in response to altered glycosylation, we investigated whether ERK1 and ERK2 phosphorylation were affected in IGF-1-treated cells (W5, Lec1, and Lec4). To accomplish this, serum-starved W5, Lec1, and Lec4 cells were stimulated with or without IGF-1LR3 (ligand for IGF-1R) for 30 min, and the levels of ERK1, ERK2, pERK1, and pERK2 were determined by Western blotting(Figure 8A,B). The band intensities for pERK1 and pERK2 were calculated together as pERK levels and ERK1 and ERK2 were combined together for ERK levels. The levels of phosphorylated forms of ERK were normalized with total ERK levels for each cell type (W5, Lec1, Lec4). In cells without stimulation, levels of pERK/ERK were reduced to 67.5% in Lec1 and 42.5% in Lec4 cells compared to W5. Interestingly, W5 cells responded with enhanced pERK/ERK levels in IGF-1-stimulated cells; however, the IGF-1 stimulation in Lec1 and Lec4 cells still reduced the levels of pERK/ERK. This suggests an independent effect of IGF-1R loss on ERK signaling (Figure 8C,D).

### 3.9. IGF1-R and IQGAP1 Interact In Silico

**Figure 9:**
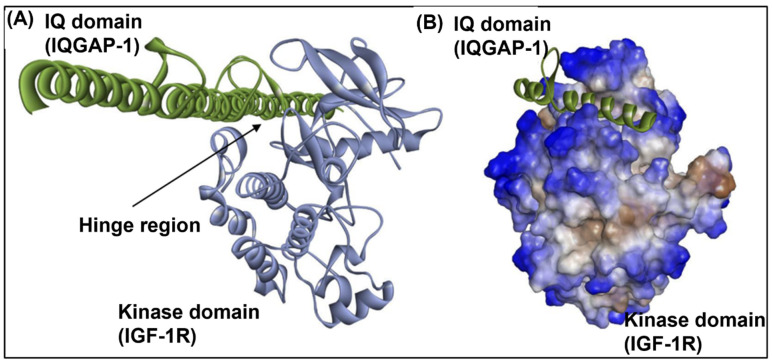
Docked poses of IQGAP1 and IGF1-R through their IQ (modeled) and kinase (PDB ID: 1JQH) domains, respectively. (**A**) represents the interaction of the IQ domain (pale green) and kinase domain (pale blue) in a cartoon view, and (**B**) represents the interaction of the IQ domain (pale green) with the hydrophobic surface (brown hydrophobic and blue hydrophilic) of the kinase domain.

IQGAP1 is a scaffold for growth factor receptor dependent activation of the MAPK cascades. We hypothesize that IQGAP1 might interact directly with the kinase domain of IGF-1R, since in vitro studies have demonstrated that EGFR has a direct association mediated through the IQ and kinase domains of IQGAP1 and EGFR, respectively. To test this hypothesis, we performed molecular docking of the IQ domain of IQGAP1 with the kinase domain of IGF-1R using three popular docking algorithms. There was a consensus in the binding sites identified by these algorithms for the top ranked docked poses (Supplementary Table S5). Based on this consensus, the N-terminal region of IQ domain interacts with the hinge region of the kinase domain (Figure 9, Supplementary Table S6). The favorable docking scores indicate a high likelihood of direct interaction between IQGAP1 and the kinase domain of IGF-1R.

## 4. Discussion

Studies have reported that glycosylation changes to the cell membrane proteins are associated with several cancers and disease conditions [68]. It is worthwhile to note that particular glycosylation forms of glycoproteins have been associated with particular cancers [69]. Our studies have revealed that differential regulation of a glycoprotein due to specific a glycoform, contributed to by Mgat1 and Mgat5 at the cell surface, may be associated with disease. Using an HR-LCMS approach, we identified 48, 44, and 43 differentially expressed proteins in W5, Lec1, and Lec4 cells, respectively, with a 1.5-fold increase to an 0.80-fold decrease at the cell surface (Figure 2A,B). Interestingly, abnormal expression of certain glycoproteins is associated with several pathological conditions. We identified a total of 15 glycoproteins in the UniProt Database (Supplementary Table S7) with a potential NX(S/T) N-glycosylation motif from the list of differentially expressed proteins at the cell surface of W5 cells. Differential glycosylation of complex glycans in membrane-bound and/or extracellular glycoproteins has clinical relevance [69,70]. Cell surface proteomics analyses of Lec1 and Lec4 cells, models of mammalian N-glycosylation deficiency, have unraveled a significant impact on the functioning of IGF-1 pathway. Lec1 and Lec4 cells have been probed previously for the glycan-processing potential of the Golgi apparatus [71].

In the current LCMS study, we found that changes in oligosaccharide structure (glycans) in Lec1 and Lec4 cells increased the amount of cell surface integrin subunit 1 (ITGB1) and decreased the amount of integrin subunit 5 (ITGB5). Targeting branched 1,6-GlcNAc structures, sialic acid, and fucose, as well as their related enzymes, in conjunction with inhibition, represents a promising therapeutic approach. We determined the effect of N-glycosylation on integrin cell surface expression via ELISA study (Figure 6C). Lec1 and Lec4 cells exhibited increased α integrins and significantly enhanced αVβ3 integrin cell surface expression. We did not observe differences in αv and β3 subunit expression in our mass spectrometry studies, which indicates that some proteins were missed in the run. Altered glycosylation of β1 integrins is prevalent in tumor cells, and is associated with cell invasiveness and metastasis [72]. Several studies have shown that altered glycosylation can affect integrin conformation [73,74]. In one of the studies investigating whether N-glycosylation is required for integrin activity, adhesion was examined in k562 cells treated with several N-glycosylation inhibitors (tunicamycin, swainsonine, or deoxymannojirimycin) [74]. The results generated in this study strongly suggest that integrin glycosylation is necessary for normal cell adhesion and spreading on integrin ligands [74]. The inhibitory effects of tunicamycin on integrin function are due to impaired trafficking of integrins through the Golgi, leading to a loss of cell surface receptors, as seen for ITGB5 in Lec1 and Lec4 cells in our study [74]. The results from our MS and ELISA study also showed a contrast finding where loss of N-glycosylation in Lec cells increased cell surface integrin expression (ITGB1, α1- α6, and αVβ3). The absence of these glycans in Lec cells did not significantly affect integrin maturation or cell surface expression for β1, checked by mass spectrometry, or α1- α6 and αVβ3, evaluated via ELISA studies. These structures (oligomannose N-glycan for Lec1 and triantennary N-glycan for Lec4) rather seem to directly modulate integrin function. Further studies are required to clarify the integrin function associated with glycosylation defects in Lec cells. We conclude at this point that dysregulation of integrins in Lec1 and Lec4 cells has been observed. Altered expression of integrins due to changes in oligosaccharide branching might contribute to the adhesive properties in glycosylated challenged cells, and these cells will give us a connection to oligosaccharide modification in cancer cells and critical proteins. N-glycosylation of collagens and laminins influences the binding to integrin receptors and promotes cancer cell adhesion [73]. The effects of Type IV collagen N-glycosylation on integrin binding have been documented in melanoma, where cell adhesion was determined by the interaction between glycosylated collagen IV and a3b1 and a2b1 integrins [75]. These data suggest that N-glycosylation of ECM proteins can regulate integrin affinity for the ligand, influence clustering, and thus result in cancer progression. Further studies are required to examine the nature of this interaction to validate whether targeting N-glycosylated ECM proteins will represent a promising therapeutic approach.

The alpha domain of IGF-1R possesses 11 potential N-glycan sites [76], which are sensitive to PNGaseF digestion in control (W5) cells (Supplementary Figure S5). We demonstrated a major defect in the processing at the membrane of IGF-1R, one of the receptor tyrosine kinases in Lec1 and Lec4 cells, through mass spectrometry analysis. We also observed downregulated expression of IGF-1R via real-time PCR (fold change = −10 for Lec1 and −5 for Lec4) and Western blot analysis (fold change = 0.50 for Lec1 and 0.14 for Lec4) (Figure 7A,B). Western blot analysis of these receptors revealed normal steady-state levels in control (W5) cells and reduced levels in Lec1 and Lec4 cells, indicating that the processing defect in these receptors is associated with their failure to reach the cell surface. The transcript levels of IGF-1R were also reduced in Lec1 and Lec4 cells, suggesting degradation of the receptor with the loss of glycosylation.

We showed that with the loss of Mgat1 and Mgat5 in CHO cells, reduced IGF-1R phosphorylation possibly inhibits activation of the MAPK pathways or specific ERK1/2 phosphorylation (Figure 8A,C). Stimulation of cultured cells (W5, Lec1, and Lec4) with 10 µg/mL IGF-1R ligand (IGF-1LR3) for 30 min reduced the cell surface IGF-1R expression and ERK signaling system. We found a significant reduction of pERK1/2 levels in Lec1 and Lec4 cells with and without stimulation (Figure 8B,C).

For IGF-1R, we observed significant reduction in Lec cells; whether this was due to degradation or internalization of the protein needs to be studied in future. In a study by Girnita et al., the inhibition of N-glycosylation using inhibitors of N-glycan biosynthesis resulted in a remarkable decrease of IGF-1R autophosphorylation together with its reduced expression at the cell surface, which was accompanied by a substantial decrease in the survival of Ewing’s sarcoma cell lines [41]. It is already known that cross-talk exists between integrin cell adhesion receptors and insulin growth factor signaling [77,78]. IGF1 interacts directly with integrins and induces integrin-IGF-IGF-1R complex formation on the cell surface [77]. The integrin-binding defective mutant of IGF1 is defective in inducing IGF signaling, although the mutant still binds to IGF-1R [79].

The present findings shed new light on the investigation of GTPase activating proteins (IQGAP1) and associated glycosylated proteins (IGF-1R). IQGAP1 is a scaffold protein that has multiple binding partners and integrates several signaling pathways to establish roles in tumorigenesis [80]. IQGAP1 has been shown to be an oncogene, where overexpression of IQGAP1 in vitro increases invasion and motility in breast cancer cells (MCF-7 and MDA-MB-231) [81]. On the other hand, knockdown of IQGAP1 reduces anchorage growth, motility, and invasion in vitro [82]. IQGAP1 binds to and modulates several growth factor receptors, including VEGFR2, FGFR1, NGFR, and EGFR [83]. Here we performed in silico docking studies, showing evidence that the IQ domain of IQGAP1 binds directly to the kinase domain of IGF-1R (Figure 9A,B). IQGAP1 binding may be necessary for proper orientation of the IGF-1R intracellular domain, thereby facilitating catalytic activity and/or enabling tyrosine kinase to access the tyrosine residues for phosphate attachment. Solving the structure of all domains of IQGAP1 and its interactions with IGF-1R, along with in vitro studies, will give us additional insights. We hypothesize that reduced expression of IGF-1R at the cell surface might decrease the half-life of IQGAP1 in N-glycosylation ablated cells (Lec1 and Lec4), thereby reducing the IQGAP1 protein in Lec1 and Lec4 cells as compared to controls. The molecular mechanism by which IGF-1R stabilizes IQGAP1 remains to be determined. IQGAP1 is an important component of trafficking. We assume that overexpression of IQGAP1 might rescue the amount of IGF-1R targeted to the plasma membrane.

KEGG enrichment analysis of the differentially expressed proteins showed that the downregulated proteins UGGT1, HSPA5, HSPA8, and HSP90AA1 are enriched in protein processing in ER, the PI3K–AKT signaling pathway, RNA transport, and the estrogen signaling pathway in Lec1 cells, and in regulating the actin cytoskeleton via focal adhesion molecules in Lec4 cells (Figure 3B). UGGT1 is a carbohydrate-dependent chaperone which facilitates proper folding and maturation of the cellular N-glycoproteome [84]. The downregulated expression of heat shock proteins HSP90AA1, HSPA5, and HSPA8 was involved in apoptosis-related protein, antigen presentation, and the estrogen signaling pathway. Studies have shown HSPA8 expression is correlated with progression of gliomas [85]. Knockdown of HSPA8, on the other hand, inhibits cell proliferation and increases apoptosis [86].

**Figure 10:**
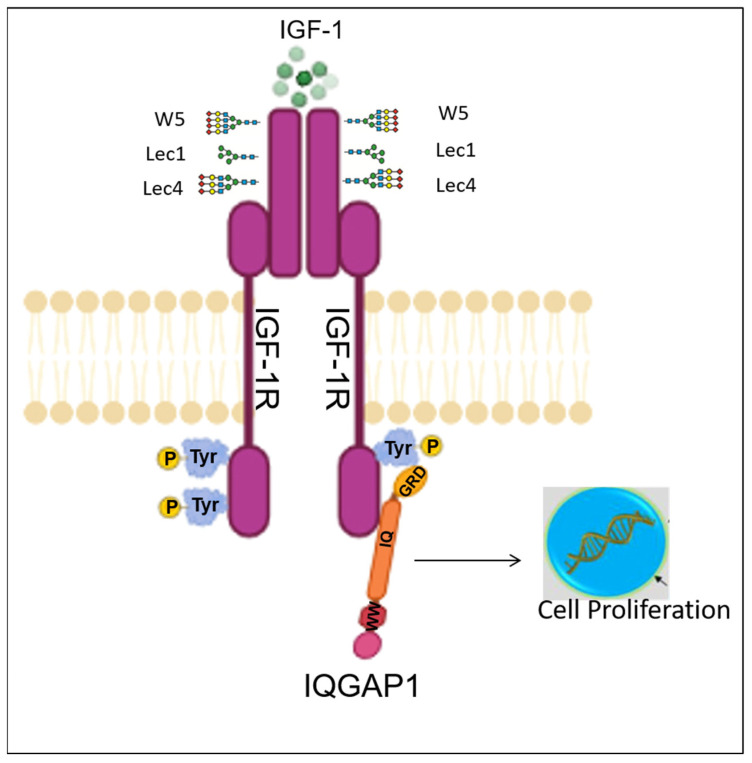
The proposed mechanism for the regulation of IGF-1R signaling and cell proliferation by the *N*-glycosylation sites. We assume that in W5 cells, IGF-1R possesses mostly complex N-glycan structures. In Lec1 and Lec4 cells, due to the loss of N-glycosylation, oligomannose N-glycans or trianteannary N-glycan structures are formed in IGF-1R. Lec1 and Lec4 cells contain limiting IGF-1R glycan branching structures which reduce cell surface localization and can turn off the IGF-1R–IQGAP1–ERK complex formation. This might result in inhibition of IGF-1R-related cellular signaling.

Based on these data, we propose a role of N-glycosylated IGF-1R function and participation in binding with IQGAP1 in inducing ERK activity. We hypothesize from this study that IQGAP1 interacts with IGF-1R and modulates IGF-1R expression and function, which might affect cell proliferation in Lec1 and Lec4 cells (Figure 10). These preliminary findings imply that IQGAP1 is a potential target for the development of additional therapeutic strategies for patients with IGF-1R defects and in glycosylation-deficient disease conditions.

## Figures and Tables

**Table 1:** Identified proteins with their accession numbers, description, q-value, peptides, and normalized abundances in W5, Lec1, and Lec4 cells are shown.

Accession	Description	q-Value	Peptides	Abundances (Grouped) W5	Abundances (Grouped) Lec1	Abundances (Grouped) Lec4
P53690	Matrix metalloproteinase-14 OS = Mus musculus OX = 10090 GN = Mmp14 PE = 2 SV = 3	0	4	21.7	280.9	62.6
P09055	Integrin beta-1 OS = Mus musculus OX = 10090 GN = Itgb1 PE = 1 SV = 1	0	10	19.1	191.5	102.3
Q71LX8	Heat shock protein 84b OS = Mus musculus OX = 10090 GN = Hsp90ab1 PE = 1 SV = 1	0	16	22.8	130.6	113.1
Q6IFX2	Keratin, type I cytoskeletal 42 OS = Mus musculus OX = 10090 GN = Krt42 PE = 1 SV = 1	0	13	47.9	173.8	100.4
A0A0R4J0I9	Low density lipoprotein receptor-related protein 1 OS = Mus musculus OX = 10090 GN = Lrp1 PE = 1 SV = 1	0	8	59.9	173.1	66.8
Q3UFT0	Uncharacterized protein (Fragment) OS = Mus musculus OX = 10090 GN = Myh9 PE = 2 SV = 1	0	14	55.4	160.8	62.3
Q3TIZ0	Tubulin alpha chain OS = Mus musculus OX = 10090 GN = Tuba1c PE = 2 SV = 1	0	16	58.9	185.6	70.8
E9QNN1	ATP-dependent RNA helicase A OS = Mus musculus OX = 10090 GN = Dhx9 PE = 1 SV = 1	0	11	79.9	85.7	86.6
Q8BTM8	Filamin-A OS = Mus musculus OX = 10090 GN = Flna PE = 1 SV = 5	0	13	50	50.2	125.1
Q3U7R1	Extended synaptotagmin-1 OS = Mus musculus OX = 10090 GN = Esyt1 PE = 1 SV = 2	0	5	45.7	47.9	205.6
A8IP69	14-3-3 protein gamma subtype OS = Mus musculus OX = 10090 GN = Ywhag PE = 1 SV = 1	0	2	185.6	185.9	25.2
A0A0A0MQF6	Glyceraldehyde-3-phosphate dehydrogenase OS = Mus musculus OX = 10090 GN = Gapdh PE = 1 SV = 1	0	6	77.8	76.9	106.7
P07901	Heat shock protein HSP 90-alpha OS = Mus musculus OX = 10090 GN = Hsp90aa1 PE = 1 SV = 4	0	11	130.8	102.9	62
P02535	Keratin, type I cytoskeletal 10 OS = Mus musculus OX = 10090 GN = Krt10 PE = 1 SV = 3	0	9	45.1	28.2	146.9
Q6P5E4	UDP-glucose: glycoprotein glucosyltransferase 1 OS = Mus musculus OX = 10090 GN = Uggt1 PE = 1 SV = 4	0	3	244.4	149.1	2.8
Q61543	Golgi apparatus protein 1 OS = Mus musculus OX = 10090 GN = Glg1 PE = 1 SV = 1	0	4	120.6	73.5	137.3
P62806	Histone H4 OS = Mus musculus OX = 10090 GN = Hist1h4a PE = 1 SV = 2	0	2	310.2	89.8	-
Q8VHY0	Chondroitin sulfate proteoglycan 4 OS = Mus musculus OX = 10090 GN = Cspg4 PE = 1 SV = 3	0	21	279.3	83.4	18.2
Q9D8N0	Elongation factor 1-gamma OS = Mus musculus OX = 10090 GN = Eef1g PE = 1 SV = 3	0	4	200.9	60.9	91.9
Q3UV17	Keratin, type II cytoskeletal 2 oral OS = Mus musculus OX = 10090 GN = Krt76 PE = 1 SV = 1	0	9	337.3	60.6	2
F8WHL2	Coatomer subunit alpha OS = Mus musculus OX = 10090 GN = Copa PE = 1 SV = 1	0	6	29.5	9	161.7
A0A2R8VHP3	Predicted pseudogene 5478 OS = Mus musculus OX = 10090 GN = Gm5478 PE = 1 SV = 1	0	9	25.1	6.3	193.7
Q6NZJ6	Eukaryotic translation initiation factor 4 gamma 1 OS = Mus musculus OX = 10090 GN = Eif4g1 PE = 1 SV = 1	0	5	229	50.6	35
Q9QXS1	Plectin OS = Mus musculus OX = 10090 GN = Plec PE = 1 SV = 3	0	53	54.5	9.8	18.1
B2RU65	Deubiquitinating enzyme 1 OS = Mus musculus OX = 10090 GN = Usp17la PE = 2 SV = 1	0.008	1	352.1	47.4	0.3
P20029	Endoplasmic reticulum chaperone BiP OS = Mus musculus OX = 10090 GN = Hspa5 PE = 1 SV = 3	0	22	134.5	18.3	155.1
E9Q557	Desmoplakin OS = Mus musculus OX = 10090 GN = Dsp PE = 1 SV = 1	0	7	178.6	29.6	133.1
Q6A0F1	MKIAA0002 protein (Fragment) OS = Mus musculus OX = 10090 GN = Cct8 PE = 2 SV = 1	0	7	199.9	29.9	58.5
P70168	Importin subunit beta-1 OS = Mus musculus OX = 10090 GN = Kpnb1 PE = 1 SV = 2	0	7	203.3	27.6	82.3
Q7TMK9	Heterogeneous nuclear ribonucleoprotein Q OS = Mus musculus OX = 10090 GN = Syncrip PE = 1 SV = 2	0	3	98.5	15.3	65.1
P17751	Triosephosphate isomerase OS = Mus musculus OX = 10090 GN = Tpi1 PE = 1 SV = 4	0.003	1	342.5	44.1	7.4
Q60751	Insulin-like growth factor 1 receptor OS = Mus musculus OX = 10090 GN = IGF-1R PE = 1 SV = 3	0.001	1	358.9	41.1	-
Q542X7	Chaperonin subunit 2 (Beta), isoform CRAa OS = Mus musculus OX = 10090 GN = Cct2 PE = 1 SV = 1	0	3	214.5	23	90
Q8BFY9	Transportin-1 OS = Mus musculus OX = 10090 GN = Tnpo1 PE = 1 SV = 2	0	1	329.1	27	17.2
Q3UCD3	Annexin OS = Mus musculus OX = 10090 GN = Anxa2 PE = 2 SV = 1	0	3	299.4	11.4	45.1
Q3TRW3	Uncharacterized protein OS = Mus musculus OX = 10090 GN = Snd1 PE = 2 SV = 1	0	7	182.6	4.8	102.3
Q3TH04	Uncharacterized protein (Fragment) OS = Mus musculus OX = 10090 GN = Hspa8 PE = 2 SV = 1	0	9	369.3	7.2	4.8
Q6ZQK2	MKIAA0051 protein (Fragment) OS = Mus musculus OX = 10090 GN = IQGAP1 PE = 2 SV = 1	0	7	307.7	1.6	28.4
B2CY77	Laminin receptor (Fragment) OS = Mus musculus OX = 10090 GN = Rpsa PE = 2 SV = 1	0	2	111.2	-	86.4
A0A1L1SV25	Alpha-actinin-4 OS = Mus musculus OX = 10090 GN = Actn4 PE = 1 SV = 1	0	3	194.8	-	88.3
G5E8F8	Integrin beta OS = Mus musculus OX = 10090 GN = Itgb5 PE = 1 SV = 1	0	4	259	-	73.3
B7ZWC0	N-myc downstream regulated gene 1 OS = Mus musculus OX = 10090 GN = Ndrg1 PE = 2 SV = 1	0	1	307.5	-	49.2
G5E866	Splicing factor 3B subunit 1 OS = Mus musculus OX = 10090 GN = Sf3b1 PE = 1 SV = 1	0	2	339.9	-	24.9
Q9DCL9	Multifunctional protein ADE2 OS = Mus musculus OX = 10090 GN = Paics PE = 1 SV = 4	0		375.4	-	10.5
Q3U900	Dolichyl-diphosphooligosaccharide--protein glycosyltransferase subunit 1 OS = Mus musculus OX = 10090 GN = Rpn1 PE = 2 SV = 1	0.005	1	273	-	12.9
Q792Z1	MCG140784 OS = Mus musculus OX = 10090 GN = Try10 PE = 1 SV = 1	0	1	391	-	4
A0JNY7	Eukaryotic translation initiation factor 4, gamma 2 OS = Mus musculus OX = 10090 GN = Eif4g2 PE = 2 SV = 1	0	1	398.2	-	0.5
P29341	Polyadenylate-binding protein 1 OS = Mus musculus OX = 10090 GN = Pabpc1 PE = 1 SV = 2	0	4	178.9	10	63.6

**Table 2:** List of upregulated and downregulated cell surface proteins in Lec1 and Lec4 cells identified by HR-LCMS.

Lec1 Cells		
Proteins	Protein Function and Activity	Regulation
MMP14	Collagenolytic activity	Upregulated
KRT42, TUBA1C	Microtubule association	Upregulated
ITGB1	Cytoskeleton and the extracellular matrix interaction.	Upregulated
HSP90AB1	Chaperone-related activity	Upregulated
LRP9	Internalization/signal transduction	Upregulated
IQGAP1	GTPase activating protein	Downregulated
IGF1-R	Cell growth proliferation and survival control	Downregulated
**Lec4 Cells**		
**Proteins**	**Protein Function and Activity**	**Regulation**
MMP14	Collagenolytic activity	Upregulated
KRT42, KRT10, TUBA1C, GM5478, FLNA	Microtubule association	Upregulated
ITGB1	Cytoskeleton and the extracellular matrix interaction.	Upregulated
HSP90AB1	Chaperone-related activity	Upregulated
COPA, EYST1	Protein transport	Upregulated
GAPDH	Glycolysis	Upregulated
IQGAP-1	GTPase activating protein.	Downregulated

**Table 3:** Primers used for validation of the genes.

Gene		Nucleotide Sequence from 5′to 3′	Product Size in bp
*IGF-1R*	Forward Reverse	GAGCCAAGACCCGAAAACTC AGTTCCCTGGGTTTAGACGG	181
*HSPA5*	Forward Reverse	TATTCCTGCGTCGGTGTGTT ATTCCAAGTGCGTCCGATGA	198
*HSPA8*	Forward Reverse	TGATCGGGCGTAGGTTTGAT CGCTTCTGCAATTTCCTTCA	202
*UGGT1*	Forward Reverse	TACGATGCCGTATTGGAAGC ACGACTTGCACCCTTCTGGT	221
*IQGAP1*	Forward Reverse	GGCAGAACGTGGCTTATGAA TTTCTTCAGGGACACCACTTTG	209
*MMP14*	Forward Reverse	CCAAGGCAGCAACTTCAGC CAAATCAGCCTTGCCTGTCA	212
*ITGB1*	Forward Reverse	AATGCCAAATCTTGCGGAGA TATGTCACTTGGCTGGCAAC	221

## Data Availability

All dataset generated for this study are included in the article/supplementary article.

## Supplementary Materials

The following supporting information can be downloaded at: https://www.mdpi.com/article/10.3390/ijms232314952/s1.
