# Supplementary material for: N-Linked Glycosylation in Chinese Hamster Ovary Cells Is Critical for Insulin-like Growth Factor 1 Signaling
Source: Int J Mol Sci. 2022 Nov 29;23(23):14952. doi: 10.3390/ijms232314952 (PMC9735751; doi:10.3390/ijms232314952)
Supplement: Supplementary file 1 [file ijms-23-14952-s001.zip › ijms-1944631-supplementary.pdf]

Supplementary Figures and Tables

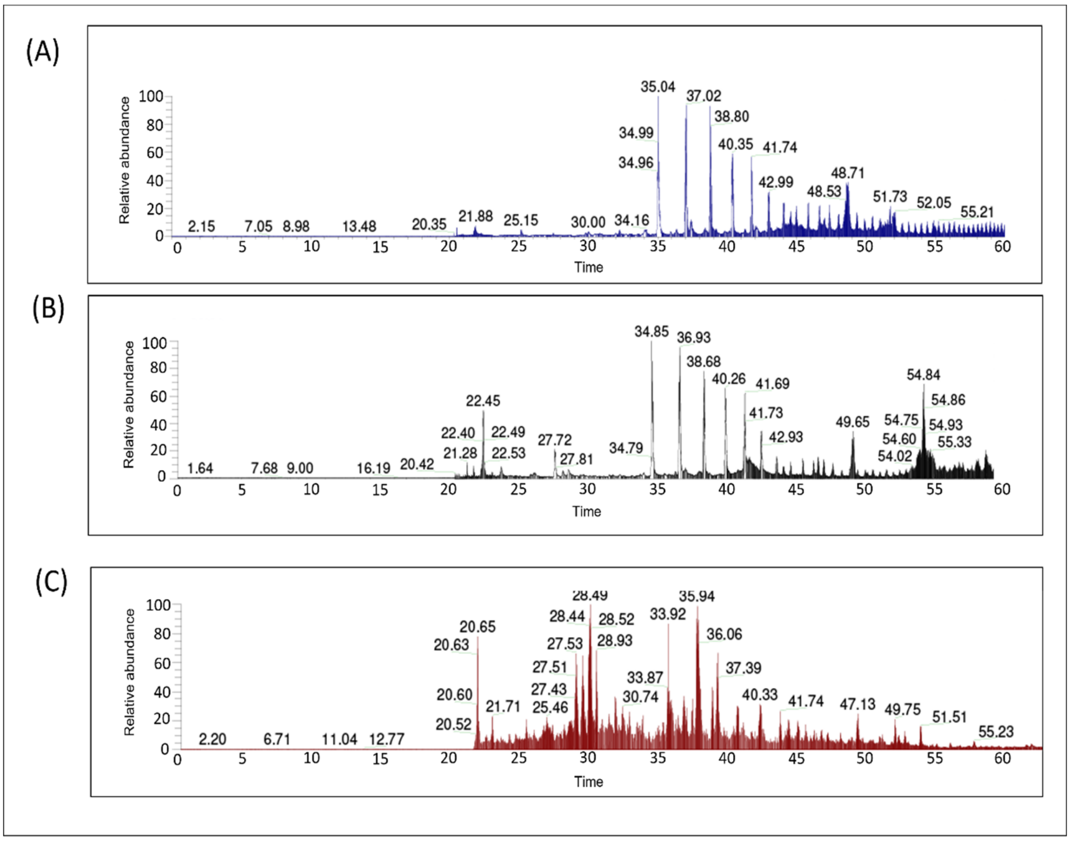

Figure S1. Base peak chromatogram of W5, Lec1, and Lec4 cells in high-frequency LCMS observation. Total ion chromatograph of cell surface proteins extracted from (**A**) W5 or CHO, (**B**) Mgat1-deficient Lec1 cells, (**C**) Mgat5-deficient Lec4 cells. Graph shows peaks of relative abundance against time.

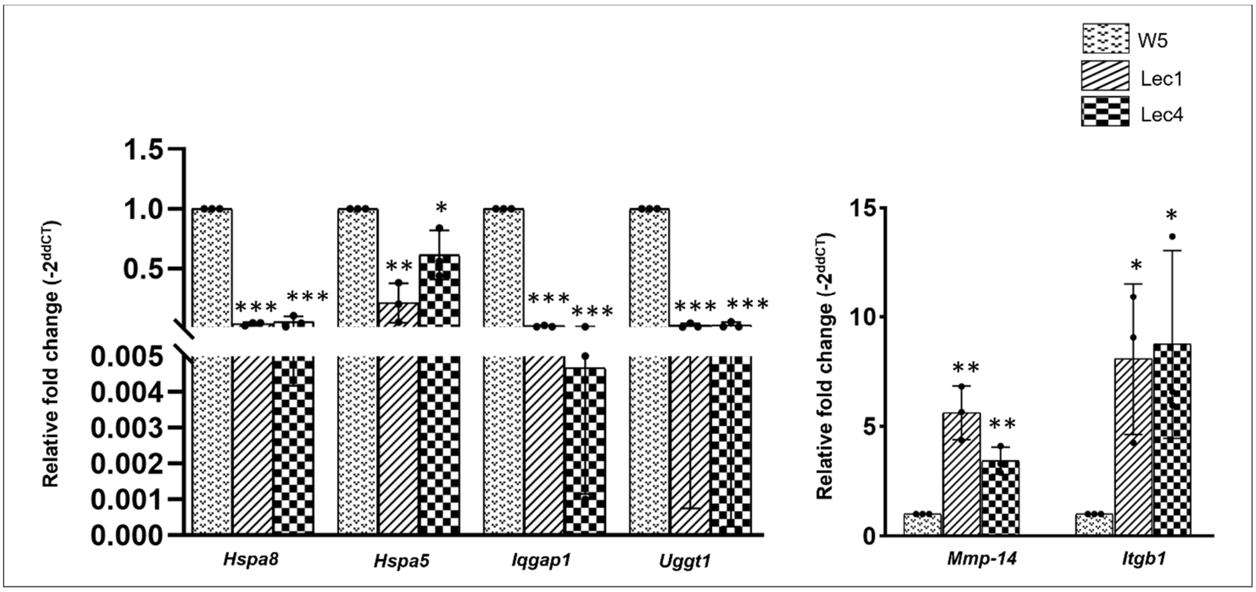

Figure S2. Real-time PCR analysis for validation of data obtained through mass spectrometry for W5, Lec1, and Lec4 cells. q-RT PCR for downregulated genes (*Hspa8*, *Hspa5*, *Uggt1*, *Iqgap1*) and upregulated genes (*Mmp14* and *Itgb1*) were quantified and normalized using housekeeping genes *(Gapdh, B-Actin), n = 3.* *** *p* value <0.001,** *p* value <0.01, and * *p* value <0.05.

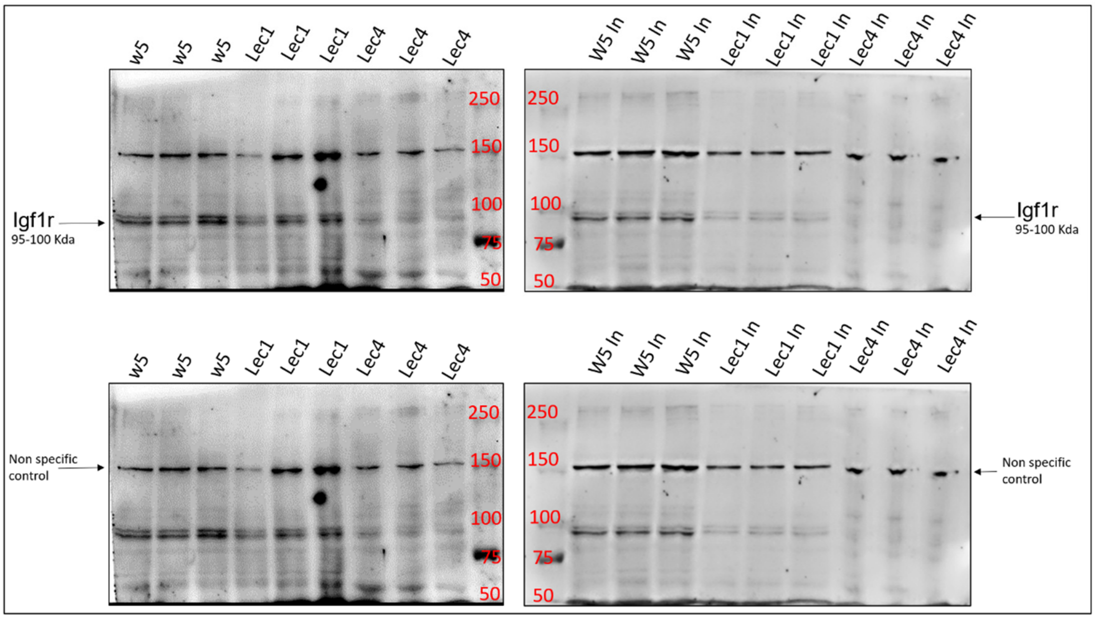

Figure S3. Full image for Western blot analysis for IGF–1R and nonspecific band in W5, Lec1, and Lec4 cells with and without stimulation by IGF–1R ligand (IGF–1LR3). Ladder is marked in red and appropriate molecular weight of the protein (IGF–1R) is shown by arrows.

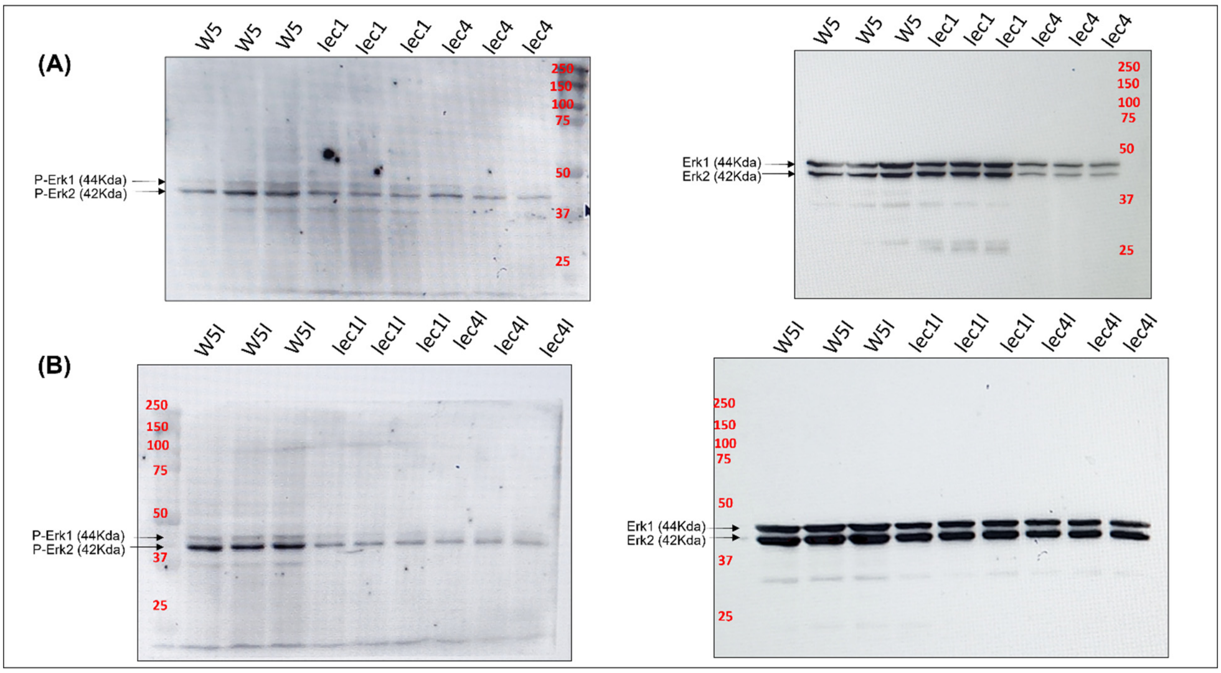

Figure S4. Full image for Western blot analysis of pERK1/2, ERK1/2 in W5, Lec1, and Lec4 cells, with noninduced cells (**A**) and cells induced with IGF–1LR3 (**B**). Ladder is marked in red and appropriate molecular weight of the protein (IGF–1R) is shown by arrows.

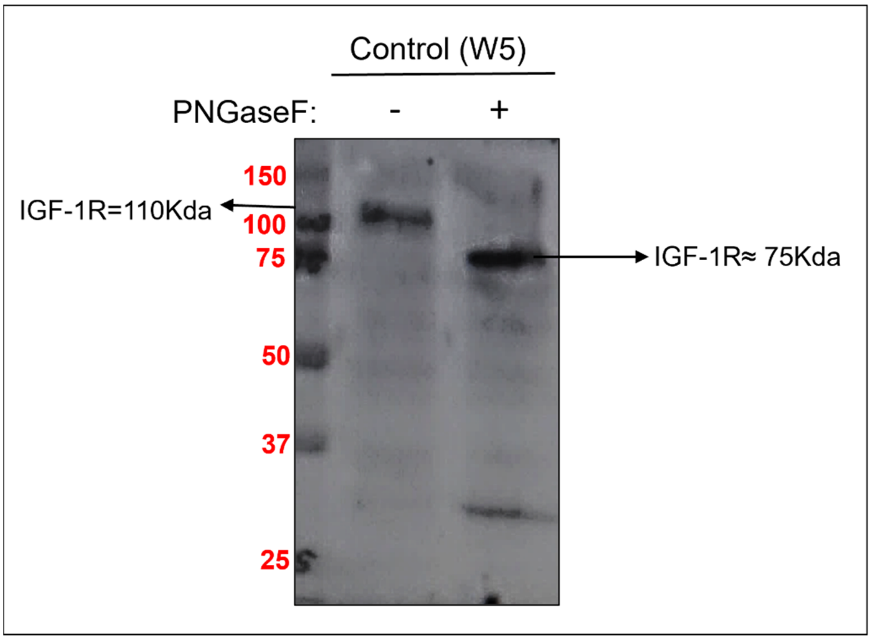

Figure S5. Western blot analysis of IGF–1R in W5 cells after PNGaseF treatment for 2 h. In untreated W5 cells, IGF-1R shows a band at 110 Kda. After treatment with PNGaseF, the IGF–1R band was reduced to approximately 75 Kda.

Supplementary Table S1. PANTHER classification of molecular function, biological process, and protein class for differentially expressed proteins in W5, Lec1, and Lec4 cells.

W5 cells		
**Molecular Function**	**No of Genes**	**Percentage**
Binding	23	47.91
Catalytic Activity	10	20.83
Molecular Function Regulator	1	2.083
Molecular Transducer Activity	1	2.083
Structural Molecular Activity	2	4.166
Translational Regulator Activity	2	4.166
Transporter Activity	2	4.166
**Lec1 cells**		
**Molecular Function**	**No of Genes**	**Percentage**
Binding	20	51.2
Catalytic Activity	10	25.6
Molecular Function Regulator	1	2.56
Molecular Transducer Activity	1	2.56
Structural Molecular Activity	2	5.12
Translational Regulator Activity	1	2.56
Transporter Activity	2	5.12
**Lec4 cells**		
**Molecular Function**	**No of Genes**	**Percentage**
Binding	22	47.8
Catalytic Activity	10	21.7
Molecular Function Regulator	1	2.17
Structural Molecular Activity	3	6.52
Translational Regulator Activity	2	4.34
Transporter Activity	2	4.34
**W5 cells**		
**Biological Process**	**No. of Genes**	**Percentage**
Biological Adhesion	3	6.25
Biological Regulation	10	20.83
Cellular Process	26	54.16
Developmental Process	3	6.25
Localization	6	12.5
Locomotion	1	2.083
Metabolic Process	11	22.91
Multicellular organismal process	3	6.25
Response to stimulus	11	22.91
Signaling	5	10.41
**Lec1 cells**		
**Biological Process**	**No. of Genes**	**Percentage**
Biological Adhesion	2	5.12
Biological Regulation	8	20.51
Cellular Process	23	58.97
Developmental Process	3	7.69

Localization	5	12.82
Locomotion	1	2.56
Metabolic Process	10	25.64
Multicellular organismal process	3	7.69
Response to stimulus	9	23.07
Signaling	3	7.69
**Lec4 cells**		
**Biological Process**	**No. of Genes**	**Percentage**
Biological Adhesion	3	6.52
Biological Regulation	9	19.56
Cellular Process	27	58.69
Developmental Process	2	4.34
Localization	5	10.86
Locomotion	2	4.34
Metabolic Process	13	28.26
Multicellular organismal process	2	4.34
Response to stimulus	9	17.56
Signaling	4	8.69
**W5 cells**		
**Protein Class**	**No of proteins**	**Percentage**
calcium-binding protein	1	2.08
cell adhesion molecule	2	4.16
Chaperone	4	8.33
cytoskeletal protein	3	6.25
membrane traffic protein	1	2.08
metabolite interconversion enzyme	3	6.25
nucleic acid metabolism protein	3	6.25
protein modifying enzyme	3	6.25
protein-binding activity modulator	1	2.08
scaffold/adaptor protein	1	2.08
transfer/carrier protein	1	2.08
translational protein	2	4.16
transmembrane signal receptor	1	2.08
transporter	2	4.16
**Lec1 cells**		
**Protein Class**	**No of Genes**	**Percentage**
calcium-binding protein	1	2.56
cell adhesion molecule	1	2.56
Chaperone	4	10.2
cytoskeletal protein	3	7.69
membrane traffic protein	1	2.56
metabolite interconversion enzyme	3	7.69
nucleic acid metabolism protein	2	5.12
protein modifying enzyme	2	5.12
protein-binding activity modulator	1	2.56
scaffold/adaptor protein	1	2.56
transfer/carrier protein	1	2.56

translational protein	1	2.56
transmembrane signal receptor	1	2.56
transporter	2	5.12
**Lec4 cells**		
**Protein Class**	**No of genes**	**Percentage**
calcium-binding protein	1	2.17
cell adhesion molecule	2	4.34
Chaperone	4	8.69
cytoskeletal protein	3	6.52
membrane traffic protein	1	2.17
metabolite interconversion enzyme	4	8.69
nucleic acid metabolism protein	3	6.52
protein modifying enzyme	4	8.69
protein-binding activity modulator	1	2.17
scaffold/adaptor protein	1	2.17
transfer/carrier protein	1	2.17
translational protein	3	6.52
transporter	2	4.34

Supplementary Table S2. KEGG pathways of differentially expressed proteins of Lec1 and Lec4 cells analyzed using DAVID tool.

**Lec1 cells**			
Kegg Pathway	Count	Percentage	*p*-value
Protein processing in Endoplasmic Reticulum	5	12.8	1.4×10^{-3}
PI3K-Akt Signaling Pathway	5	12.8	1.9×10^{-2}
Proteoglycans in Cancer	4	10.3	2.2×10^{-2}
Antigen Processing and Presentation	3	7.7	2.5×10^{-2}
Progesterone mediated oocyte maturation	3	7.7	2.7×10^{-2}
Prostate Cancer	3	7.7	2.8×10^{-2}
Estrogen Signaling Pathway	3	7.7	3.4×10^{-2}
RNA Transport	3	7.7	9.1×10^{-2}
**Lec4 cells**			
Kegg Pathway	Count	Percentage	*p*-value
Protein processing in Endoplasmic Reticulum	6	13.0	3.6×10^{-4}
RNA transport	4	8.7	2.5×10^{-2}
Arrhythmogenic right ventricular cardiomyopathy (ARVC)	3	6.5	2.6×10^{-2}
Antigen Processing and Presentation	3	6.5	3.8×10^{-2}
Proteoglycans in Cancer	4	8.7	4.0×10^{-2}
PI3K-Akt Signaling Pathway	5	10.9	4.1×10^{-2}
Focal Adhesion	4	8.7	4.2×10^{-2}
Regulation of Actin cytoskeleton	4	8.7	4.5×10^{-2}
Estrogen Signaling Pathway	3	6.5	5.2×10^{-2}

Supplementary Table S3. Enriched gene sets identified by GSEA analysis.

**Lec1 cells**			
**Name**	**Genes**	**NES**	**FDR q value**

Insulin IGF pathway-protein kinase B signaling cascade	*IGF-1R*	−1	1
Glycolysis	*TPI1, GAPDH*	−1	1
Parkinson disease	*HSPA8, HSPA5, YWHAG*	−1	1
Alzheimer's disease-presenilin pathway	*MMP14, LRP1*	1	1
Nicotinic acetylcholine receptor signaling pathway	*MYH9*	1	1
Inflammation mediated by chemokine and cytokine signaling pathway	*MYH9, ITGB1*	1	1
Huntington's disease	*GAPDH*	1	1
EGF receptor signaling pathway	*YWHAG*	1	1
Nicotine pharmacodynamics pathway	*FLNA*	1	1
PNAT	*FLNA*	1	1
Insulin IGF Pathway-Mitogen activated protein kinase kinase map kinase cascade	*IGF-1R*	−1	1
Cytoskeletal regulation by Rho GTPase	*MYH9*	1	1
Integrin signaling pathway	*FLNA, ITGB1*	1	1
Apoptosis signaling pathway	*HSPA8, HSPA5*	−1	1
FGF signaling pathway	*YWHAG*	1	1
**Lec4 cells**			
**Name**	**Genes**	**NES**	**FDR q value**
Glycolysis	*TPI1, GAPDH*	0.9156	1
Parkinson disease	*HSPA8, HSPA5, YWHAG*	−1	1
Alzheimer's disease-presenilin pathway	*MMP14, LRP1*	1	1
Nicotinic acetylcholine receptor signaling pathway	*MYH9*	1	1
Inflammation mediated by chemokine and cytokine signaling pathway	*MYH9, ITGB1*	1	1
Huntington's disease	*GAPDH*	1	1
EGF receptor signaling pathway	*YWHAG*	−1	1
Nicotine pharmacodynamics pathway	*FLNA*	1	1
PNAT	*FLNA*	1	1
Cytoskeletal regulation by Rho GTPase	*MYH9*	1	1
Integrin signaling pathway	*FLNA, ITGB1, ITGB5, ACTN4*	1	1
Apoptosis signaling pathway	*HSPA8, HSPA5*	−1	1
FGF signaling pathway	*YWHAG*	−1	1

Supplementary Table S4. Summary of top associated networks in Lec1 and Lec4 cells following Ingenuity Pathway Analysis (IPA).

**Lec1 cells**		
**Associated Network Function**	**Score**	**Focus Molecules**
Protein Degradation, Protein Synthesis, Post-translational Modification	28	17
Cellular Assembly and organization, Cellular Function and Maintenance, Embryonic Development	25	12
Cell-to-Cell signaling and Interaction, Cellular Movement, Cell Death and Survival	17	7
Cell Death and Survival, Cellular Function and maintenance, Hematological system Development and Function	12	4

Lec4 cells		
**Associated Network Function**	**Score**	**Focus Molecules**
Protein Synthesis, Post translational Modification, Protein Folding	38	18
Cellular Movement, Cell-to-Cell signaling and Interaction, Cellular Assembly and organization	35	15
Cellular Development, Cellular Growth and Proliferation, Tumor Development	26	9
Cell Death and Survival, Cellular Function and maintenance, Hematological system Development and Function	3	5

Supplementary Table S5. Docked scores of three algorithms for IQ domain of IQGAP1 and kinase domain of IGF-1R.

ZDOCK		ClusPro		FireDock	
**Pose**	**ZRank Score**	**Cluster**	**Weighted Score**	**Rank**	**Global Energy**
1	−118.0 *	0	−925.8	1	−44.8 *
2	−115.1	1	−1015.4 *	2	−35.0
3	−111.4	2	−980.8	3	−34.0
4	−103.9	3	−933.8	4	−32.1
5	−103.6	4	−921.5	5	−25.7

* The binding site of this pose is common for the three algorithms.

Supplementary Table S6. Intermolecular interactions observed in top docked pose identified by ZDOCK.

Types of Interaction	Interacting Residues	
	**IQ Domain (IQGAP1)**	**kinase Domain (IGF-1R)**
Hydrogen Bonds	PHE771	LYS1023
	ILE779	ARG1064
	TRP786, LYS793	ASP1146
	LYS792	GLU1276
Electrostatic interaction	LYS790	ASP1146
	LYS793	GLU1118, GLU1271
	ASP797	LYS1111
Hydrophobic interaction	TYR789	VAL1277

Supplementary Table S7. Identified glycoproteins with there accession numbers; descriptions are shown.

Accession	Description
Q6P5E4	UDP-glucose:glycoprotein glucosyltransferase 1 OS = Mus musculus OX = 10090 GN = Uggt1 PE = 1 SV = 4
G5E8F8	Integrin beta OS = Mus musculus OX = 10090 GN = Itgb5 PE = 1 SV = 1
F1LS79	Chondroitin sulfate proteoglycan 4 OS = Rattus norvegicus OX = 10116 GN = Cspg4 PE = 4 SV = 1
Q61543	Golgi apparatus protein 1 OS = Mus musculus OX = 10090 GN = Glg1 PE = 1 SV = 1
O35112	CD166 antigen OS = Rattus norvegicus OX = 10116 GN = Alcam PE = 1 SV = 1
Q63617	Hypoxia up-regulated protein 1 OS = Rattus norvegicus OX = 10116 GN = Hyou1 PE = 1 SV = 1
P09055	Integrin beta-1 OS = Mus musculus OX = 10090 GN = Itgb1 PE = 1 SV = 1
P27046	Alpha-mannosidase 2 OS = Mus musculus OX = 10090 GN = Man2a1 PE = 1 SV = 2
Q9ERF7	Intercellular adhesion molecule 1 OS = Cricetulus griseus OX = 10029 GN = ICAM1 PE = 2 SV = 1
P97629	Leucyl-cystinyl aminopeptidase OS = Rattus norvegicus OX = 10116 GN = Lnpep PE = 1 SV = 1
E9QB01	Neural cell adhesion molecule 1 OS = Mus musculus OX = 10090 GN = Ncam1 PE = 1 SV = 2
B2RXS4	Plexin-B2 OS = Mus musculus OX = 10090 GN = Plxnb2 PE = 1 SV = 1
G3V679	Transferrin receptor protein 1 OS = Rattus norvegicus OX = 10116 GN = Tfrc PE = 1 SV = 1
P12382	ATP- dependent 6-phosphofructokinase, liver type OS = Mus musculus OX = 100
